# Identification of 12 OCA Cases in Chinese Population and Two Novel Variants

**DOI:** 10.3389/fgene.2022.926511

**Published:** 2022-07-12

**Authors:** Zilin Zhong, Zheng Zhou, Jianjun Chen, Jun Zhang

**Affiliations:** ^1^ Birth Defect Group, Translational Research Institute of Brain and Brain-Like Intelligence, Shanghai Fourth People’s Hospital, School of Medicine, Tongji University, Shanghai, China; ^2^ Department of Regenerative Medicine, School of Medicine, Tongji University, Shanghai, China; ^3^ National Laboratory of Biomacromolecules, Institute of Biophysics, Chinese Academy of Sciences, Beijing, China; ^4^ University of Chinese Academy of Sciences, Beijing, China; ^5^ Department of Medical Genetics, School of Medicine, Tongji University, Shanghai, China; ^6^ Research Center for Translational Medicine, Shanghai East Hospital, School of Medicine, Tongji University, Shanghai, China; ^7^ Key Laboratory of Spine and Spinal Cord Injury Repair and Regeneration of Ministry of Education, Stem Cell Translational Research Center of Tongji Hospital, School of Medicine, Tongji University, Shanghai, China

**Keywords:** albinism, TYR, OCA2, variant, structure

## Abstract

OCA (oculocutaneous albinism) refers to a group of heterogeneous congenital disorders of which the common manifestations are variable degrees of cutaneous hypopigmentation and significant visual impairment, including poor visual acuity, photophobia, and nystagmus. Molecular analysis may elucidate its pathogenesis and be in favor of accurate diagnosis. High-throughput sequencing and Sanger sequencing were performed to detect mutational alleles and *in silico* analysis was performed for prediction of variant pathogenicity. Ten *TYR*-related and two *OCA2*-related patients were identified with 16 different variants with potential pathogenicity. Two novel missense variants [*TYR*: c.623T > G, *p*(Leu208Arg) and *OCA2*: c.1325A > G, *p*(Asn442Ser)] are identified in this study, and three OCA cases are reported for the first time in Chinese population based on their associated variants. Analysis of crystal structures of TYR ortholog and its paralog TYRP1 suggests that the substitution of Leu^208^ may have an impact on protein stability. This study may facilitate OCA diagnosis by expanding the mutational spectrum of *TYR* and *OCA2* as well as further basic studies about these two genes.

## Introduction

OCA (oculocutaneous albinism) is a group of congenital pigmentary disorders which result from the deficiency of melanin predominantly responsible for skin, hair, and iris color and providing us thermoregulation and photoprotection. Such patients clinically present a series of hallmark manifestations including variable degrees of cutaneous hypopigmentation, poor visual acuity, nystagmus, and photophobia. It is inherited in the autosomal recessive pattern and has a prevalence of about 1:18,000 in the Chinese population (Gong et al., 1994; Federico and Krishnamurthy, 2021). OCA can be either nonsyndromic or syndromic, and molecular testing is helpful in its accurate diagnosis and health care (Federico and Krishnamurthy, 2021; Pennamen et al., 2021).

Tyrosinase (E.C. 1.14.18.1) encoded by *TYR* is the rate-limiting enzyme critical for melanin synthesis, and the optimal pH condition of TYR activity is neutral (Fitzpatrick et al., 1950). The complete or partial loss of tyrosinase or its activity can lead to melanin deficiency. OCA1 (MIM#203100w), caused by variants of *TYR* (NM_000372.5) encoding a 529aa (amino acids) protein, has been reported as the most prevalent OCA in China, America, and Europe (Hutton and Spritz, 2008; Wei et al., 2010; Lasseaux et al., 2018; Marti et al., 2018; Zhong et al., 2019). *TYR* has five exons, and six pairs of primers are enough to screen its coding regions plus flanking splicing sites (Zhong et al., 2019). Thus, we used direct sequencing *TYR* helpful when considering its costeffectiveness. Tyrosinase is conserved across a broad variety of species. Crystal structures of tyrosinase from germs and its paralog TYRP1 (tyrosinase-related protein have been resolved and contribute to the prediction of variant pathogenicity. Another important protein in melanin synthesis is melanosomal transmembrane *p* protein involved in the regulation of melanosomal pH critical to TYR activity (Rinchik et al., 1993; Bellono et al., 2014). OCA2 caused by variants of OCA2 (NM_000275.3), encoding an 838aa-sized glycoprotein, is the second prevalent type in the Chinese cohort and in the European set, while being the most common worldwide due to high prevalence of 1/3,900 in sub-Saharan Africa (Lasseaux et al., 2018; Marti et al., 2018; Zhong et al., 2019; Federico and Krishnamurthy, 2021). OCA2 spans 334 kb in genomic DNA, has 25 exons, and no mutational hotspot of this gene has been observed for OCA cases (Lasseaux et al., 2018; Zhong et al., 2019; Federico and Krishnamurthy, 2021). In addition to these two genes, there are more than ten known causative OCA genes (TYRP1, SLC45A2, SLC24A5, LRMDA, MITF, HPS1, AP3B1, HPS3, HPS4, BLOC1S3, HPS5, HPS6, DTNBP1, BLOC1S6, AP3D, BLOC1S5, GPR143, LYST, MY5A, RAB27A, and MLPH) (Federico and Krishnamurthy, 2021), although their related OCA is relatively rare. Therefore, high-throughput sequencing is more effective for detection of variants in these genes. There is hitherto no resolved crystal structure of *p* protein in any species, but recently a few reports about its function may be helpful as a clue to prediction of variant pathogenicity.

In this study, all exons of *TYR* and their flanking regions were directly sequenced in all 12 patients, and then high-throughput sequencing of known OCA genes was performed on the patients without likely pathogenic variants in *TYR*. *In silico* resources about the genes and cases were reviewed to predict the pathogenicity of novel variants and speculate the possible role of the residues where novel variants change.

## Materials and Methods

### Study Cases

This study was approved by the institutional review board at Shanghai Fourth Peoples Hospital, School of Medicine, Tongji University, China. All procedures in this study were performed according to the principles of the Declaration of Helsinki. Twelve unrelated patients were recruited for this study. All the patients present obvious hypopigmentation in skin and hair and have no complaints of disorders in other organs. About 2 ml of peripheral blood was collected from the participants or their guardians who signed informed consent.

### Variants Detection and *In Silico* Analysis

Touchdown PCR amplification was carried out with the procedures of an annealing temperature of 60–57°C for all primers used in this study. Sanger sequencing was performed on total genomic DNA from all the patients with six pairs of primers previously described to amplify *TYR* exons plus their flanking regions which are mutational hotspots in Chinese OCA (Zhong et al., 2019). For the remaining unsolved patients with the abovementioned primers, TES (target exome sequencing) was carried out. About 3 micrograms of genomic DNA were quantified with a NanoDrop Spectrophotometer. Libraries were prepared according to Illumina standard protocol and were loaded on the NextSeq500 platform. Known OCA genes were included in the TES panel. SOAPaligner was used to align the reads to reference sequences of humans. The following workflow was used to filter out candidate variants. Variants in known OCA genes→ rare or absent in healthy controls→ homozygous or compound heterozygous variants. The pathogenicity of variants was predicted by *in silico* tools SIFT, Polyphen-2, and MutationTaster (Kumar et al., 2009; Adzhubei et al., 2010; Schwarz et al., 2010). Sanger sequencing was performed to verify candidate variants or for cosegregation analysis if DNA from family members was available. In addition, as for variants closed together, amplicons containing the variants were cloned into vectors and the vectors were transformed into *Escherichia coli* for amplification. Then, at least five single clones were sequenced by Sanger sequencing with the primers (M13F: 5′-GTA​AAA​CGA​CGG​CCA​GT-3′ and M13R: 5′-CAG​GAA​ACA​GCT​ATG​AC-3′) to assess genetic phase of neighboring heterozygous variants. For literature review, keywords such as Chinese, albinism, tyrosinase, OCA2, or their aliases were selected for combination and used to search in PubMed, and then duplicates were removed. All extracted data were double-checked for accuracy. All Chinese *TYR*-related or *OCA2*-related cases from the published literature (till 31 October 2021) at PubMed and its linked journal websites were reviewed to evaluate the impact of the variants identified in this study sharing on proband counts. The frequency of the variants in healthy controls of gnomAD (genome Aggregation Database) is shown in Supplementary Table S1, and the recurrence of variants in Chinese OCA cases is shown in Table S2. Evidence of pathogenicity can be categorized into very strong (PVS), strong (PS), moderate (PM), and supporting (PP) based on the guidelines of the American College of Medical Genetics and Genomics (Richards et al., 2015). The crystal structures of *Streptomyces castaneoglobisporus* tyrosinase (PDB: 1WX2) and human TYRP1 (PDB: 5M8L) were available (Matoba et al., 2006; Lai et al., 2017) and human *p* protein (PDB: AF-Q04671-F1-model_v2) was obtained from AlphaFold, and those structures were analyzed by Pymol. In addition to the abovementioned protein sequences, the following sequences *Homo sapiens* (NP_000363.1), Bos taurus (NP_851344.1), Canis lupus familiaris (NP_001002941.1), Gallus gallus (NP_989491.1), Rattus norvegicus (NP_001101005.1), Mus musculus (NP_035791.1), Xenopus tropicalis (NP_001096518.1), Danio rerio (NP_571088.3), *Homo sapiens* (NP_000266.2), Sus scrofa (NP_999259.2), Rattus norvegicus (NP_001258422.1), and Mus musculus (NP_068679.1) were used for amino acid alignment.

## Result

### Clinical Manifestation

**FIGURE 1:**
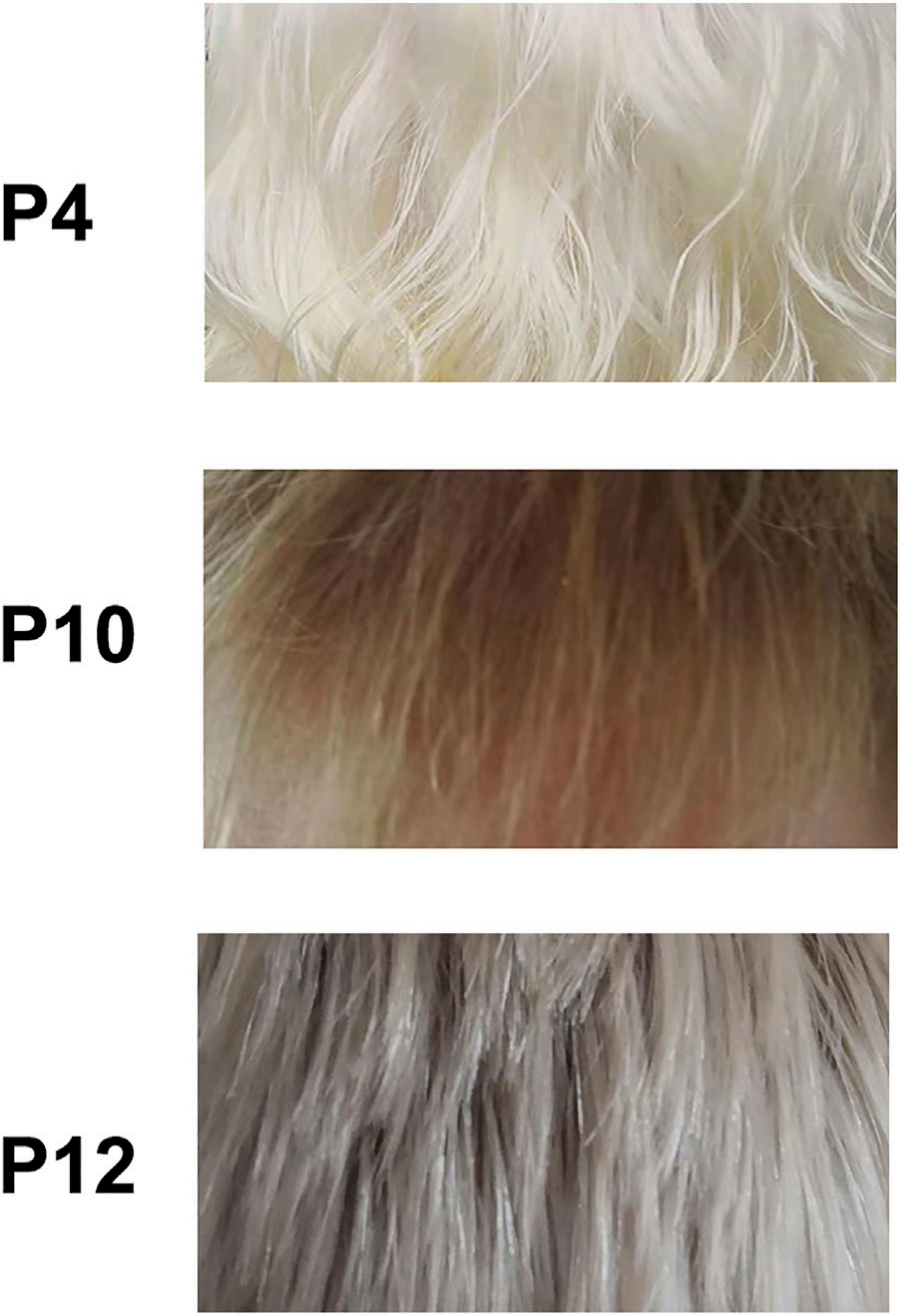
Hair phenotype of OCA cases. *p*, patient. P4 had homozygous variant *TYR*: c.623T > G, *p* (Leu208Arg). P10 had compound variants *TYR*: c.929dupC, *p* (Arg311Lysfs*7) and *TYR*: c.1037–7A > T plus c.1037–10_11delTT. P12 had compound variants *OCA2*: c.1325A > G, *p* (Asn442Ser) and *OCA2*: c.2180T > C, *p*(Leu727Pro).

In this study, 12 patients were from unrelated families. All of them have poor visual acuity, conspicuous hypopigmentation in skin and hair, and no complaint of other disorders. Their parents were non-consanguineous. Nine patients (P1–P9) exhibit stark white skin and white hair at analysis substantially without change in later age (Supplementary Table S2). The other three patients (P10–P12) present creamy white skin, and the patient P12 has freckles on the face. P10 manifests blond hair at analysis (Figure 1) and nearly no change in cutaneous color from birth to adulthood. P11 presents golden hair at analysis and light yellow hair at birth. In P12, his hair is grayish white at birth and flaxen hair at analysis (Figure 1).

### *TYR* Variants in OCA Patients

**FIGURE 2:**
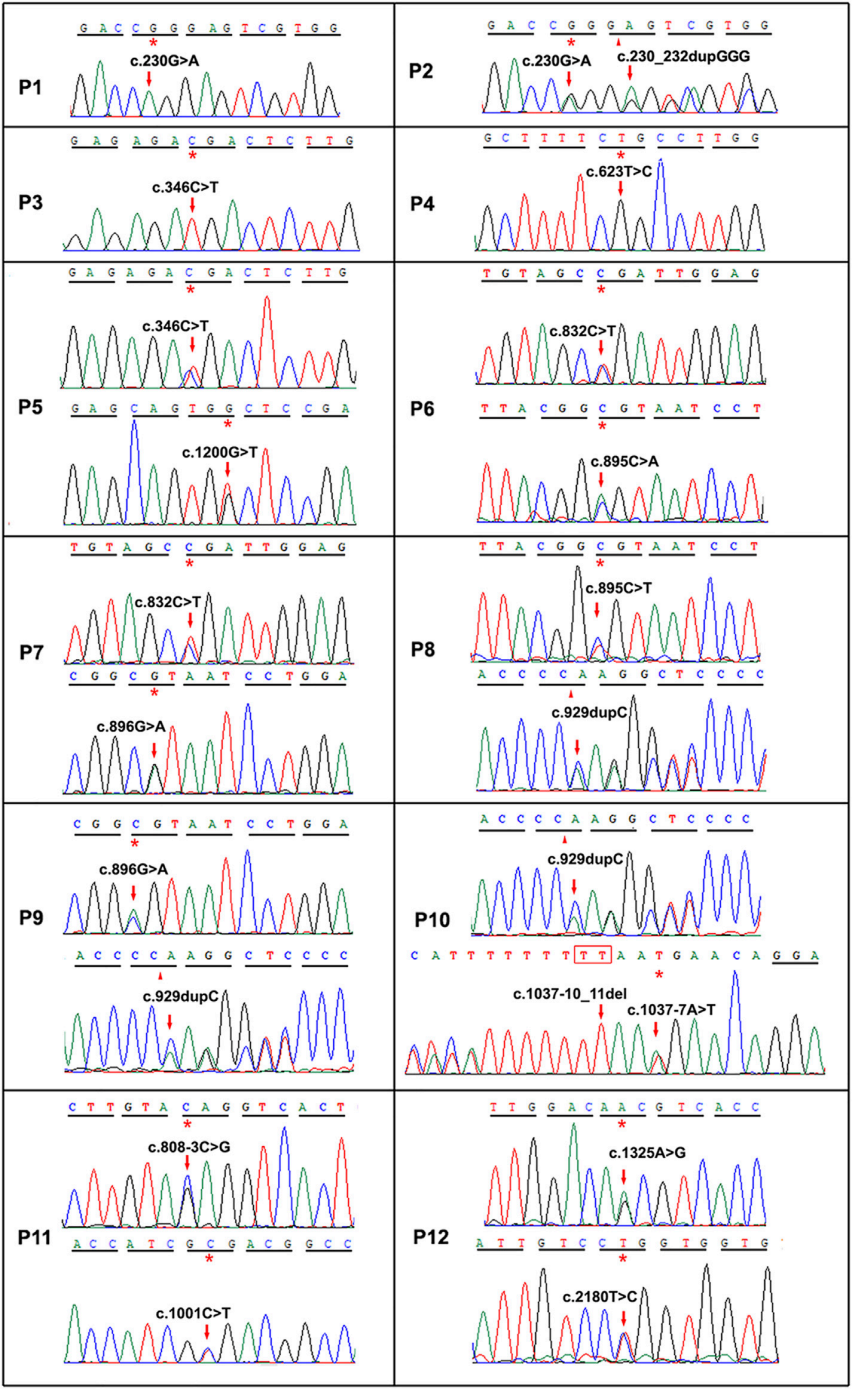
Sequence chromatograms of *TYR* or *OCA2* variants of twelve OCA cases in this study. The nucleotide changes were pointed by red arrows. *p*, patient. The first ten cases P1–P10 related to *TYR* variants, and the last two cases P11–P12 related to *OCA2*.

**FIGURE 3:**
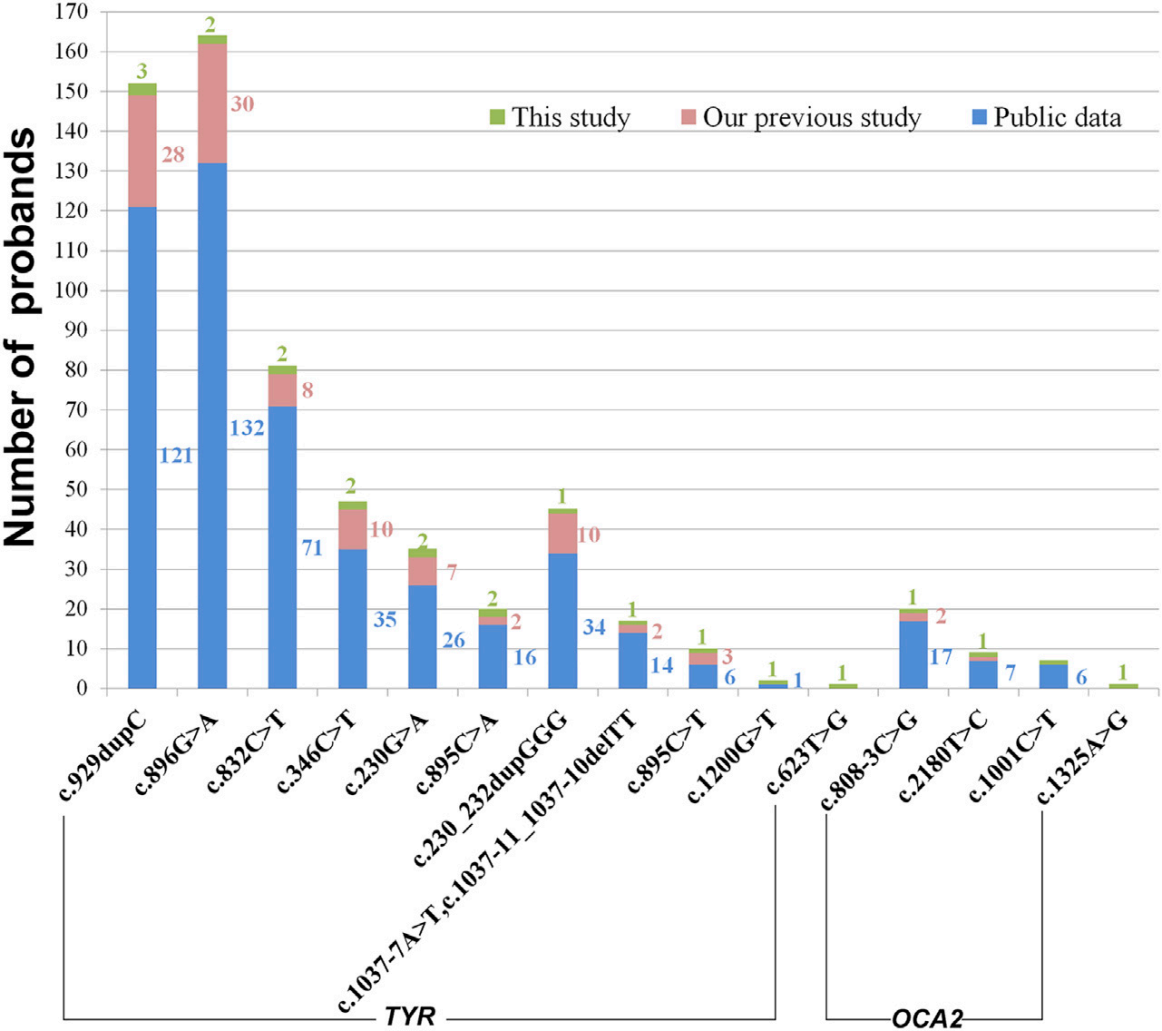
Variant sharing on proband counts. The number of probands was in the same color as that of bars. Publicly available data were collected from the published literature about Chinese OCA cases.

**FIGURE 4:**
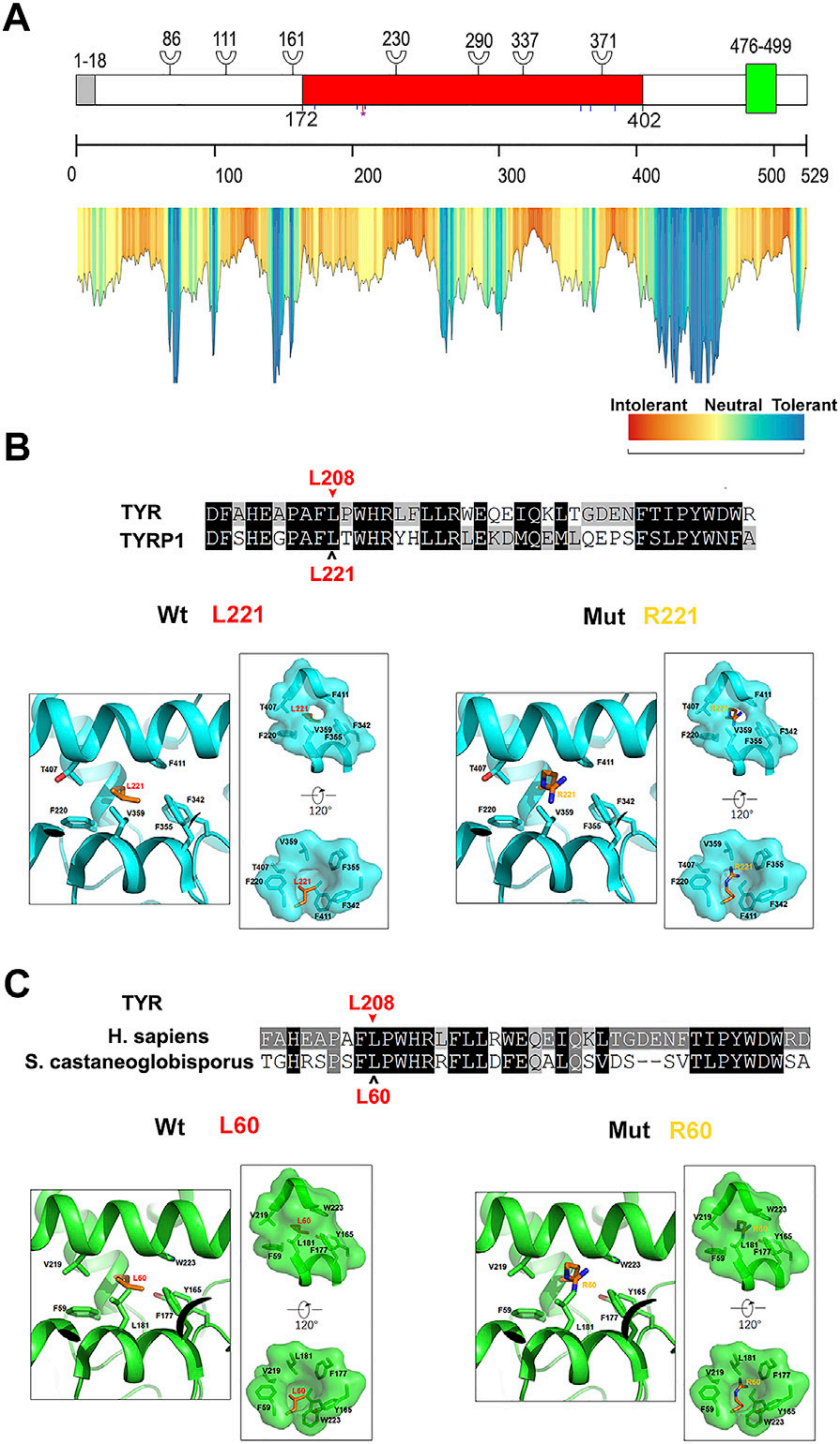
Computational analysis of the residue where a novel variant changes in TYR **(A)** Schematic representation and tolerance landscape of TYR. The signal peptide (1–18aa) was colored in gray; the common central domain of tyrosinase (172–402aa) in red; transmembrane domain (476–499aa) in green. Branch symbols indicate putative N-linked glycosylation sites. Blue lines indicate the positions of copper-coordinating histidine. The red line plus a purple blob indicate the novel variant in this study **(B)** Counterpart of TYR L^208^ in human TYRP1 and the effect of substitution of L208R. The counterpart of TYR L^208^ is L^221^ in human TYRP1. The alignment of amino acids of human TYR and its paralog TYRP1 shows that they are homologs to each other and the residue where a variant changes is located in the conserved domain. In the structure model of human TYRP1, the residue in red is the counterpart of L^208^ in human TYR, and other residues associated with it are in black. Left: structure model of wildtype TYRP1. The left square shows that L^221^ is buried in the hydrophobic pocket formed by hydrophobic side-chains of surrounding residues. Residues provided by the hydrophobic surface are highlighted, and right rectangle, the hydrophobic surface is shown in surface mode. Right: destabilization effect of leucine to arginine substitution structural analyses show that L221R causes steric hindrance and introduces changes that are incompatible with the hydrophobic environment **(C)** Counterpart of TYR L^208^ in tyrosinase from *Streptomyces castaneoglobisporus* and the effect of substitution L208R. The counterpart of TYR L^208^ is L^60^ in tyrosinase from *Streptomyces castaneoglobisporus*. Based on the alignment of amino acid sequence, the residue in TYR where a novel variant changes is in red and pointed by a red arrowhead, and its counterpart is in red and pointed by a black open arrowhead. Left: structure model of wildtype tyrosinase from *Streptomyces castaneoglobisporus*. The left square shows that L^60^ is buried in the hydrophobic pocket formed by hydrophobic side-chains of surrounding residues. Residues providing the hydrophobic surface are highlighted, and right rectangle, the hydrophobic surface is shown in surface mode. Right: destabilization effect of the substitution of leucine to arginine structural analyses show that L60R cause steric hindrance and introduces charges that are incompatible with a hydrophobic environment.

Twelve different variants of *TYR* were identified in ten OCA patients by Sanger sequencing including a homozygous variant c.623T > G in P4 (Figure 2). Nine patients had compound heterozygous variants, including P2 with c.230G > A and c.230_232dupGGG confirmed to locate on different alleles by M13 sequencing of the clone (Supplementary Figure S1). In the healthy control database, all of these variants were rare or absent, and their allele and genotype frequency data were absent (Supplementary Table S1). Moreover, c.623T > G is novel, c.1200G > T was previously reported in a Chinese patient, and other eight variants in this study have been reported in no less than four different OCA cases according to the published literature (Supplementary Table S2 and Figure 3). Except for a novel missense variant c.623T > G and the recurrent variants including the missense variant c.1200G > T, and the splicing variant c.1037–7A > T plus c.1037–10_11delTT closed together on the same allele confirmed by M13 clone sequencing, other variants have been assigned pathogenic or likely pathogenic in ClinVar, including two nonsense variants (c.346C > T and c.832C > T), a frameshift insertion c.929dupC, an in-frame insertion c.230_232dupGGG, and three missense variants (c.895C > A, c.895C > T and c.896G > A) (Table 1). The variants c.623T > G and c.1200G > T were predicted deleterious by *in silico* tools (Table 1). The variant c.1200G > T may bring about cysteine for Trp^400^ where either c.1198T > A or c.1199G > T change recorded pathogenic or likely pathogenic in ClinVar (Table 1), and thus it can be determined to be likely pathogenic (PS1, PM2, *p*P3). The novel variant c.623T > G may lead to substitution for Leu^208^ located in the mutational hotspot (exon1 and exon2) and in the region where the methadone tolerance landscape of TYR demonstrated that nearly no tolerant region is located (Wiel et al., 2019) (Figure 4A). Also, so we considered the variant c.623T > G in P4 as VUS (a variant of uncertain significance) (PM1, PM2, and *p*P3), and this patient was not detected to have candidate variants in other known OCA genes by TES. For the splicing variant, the published study has confirmed the mutational allele c.1037–7A > T plus c.1037–10_11delTT as a pathological splicing site by *in vitro* splicing assay (Goto et al., 2004). Therefore, the variant c.1037–7A > T plus c.1037–10_11delTT can be considered likely pathogenic (PS3, PM2, and *p*P5).

**TABLE 1:** Pathogenicity prediction of OCA-related variants in this study and their relevant variants.

Gene	Accession		Variant			Pathogenicity prediction in protein level			ClinVar/NCBI	
		The position where the variants change	Amino acid change	Nucleoid change	Type	Polyphen-2	SIFT	Mutation Taster	Clinical significance	Accession
*TYR*	NM_000372.5	—	*p*.Arg116*	c.346C > T	Nonsense	-	-	-	Pathogenic	VCV000099565
			*p*.Arg278*	c.832C > T	Nonsense	-	-	-	Pathogenic	VCV000099583
			*p*.Arg311Lysfs*7	c.929dupC	Indel.	-	-	-	Pathogenic	VCV000003771
			-	c.1037–7A > T, c.1037–10_11delTT	Splicing	-	-	-	NA	NA
		77_	*p*.Arg77_Glu78insGly	c.230_232dupGGG	Indel.	-	-	DC	Likely pathogenic	VCV000099554
		77	*p*.Arg77Gln	c.230G > A	Missense	PDa	DA	DC	Pathogenic/Likely pathogenic	VCV000003776
		299	*p*.Arg299Cys	c.895C > T	Missense	PDa	DA	DC	Pathogenic	VCV001284359
			*p*.Arg299Ser	c.895C > A	Missense	PDa	DA	DC	Pathogenic	VCV000099587
			*p*.Arg299His	c.896G > A	Missense	PDa	DA	DC	Pathogenic	VCV000003796
		400	*p*.Trp400Leu	c.1199G > T	Missense	PDa	DA	DC	Pathogenic/Likely pathogenic	VCV000099541
			*p*.Trp400Arg	c.1198T > A	Missense	PDa	DA	DC	Likely pathogenic	VCV000627597
			*p*.Trp400Cys	c.1200G > T	Missense	PDa	DA	DC	NA	NA
		208	*p*.Leu208Arg	**c.623T > G** ^**†**^	Missense	PDa	DA	DC	NA	NA
*OCA2*	NM_000275.3	—	-	c.808–3C > G	Splicing	-	-	-	NA	NA
		334	*p*.Ala334Val	c.1001C > T	Missense	PDa	DA	DC	Pathogenic	VCV000000958
		442	*p*.Asn442Ser	**c.1325A > G** ^**†**^	Missense	PDa	DA	DC	NA	NA
		442_	*p*.Asn442del	c.1324_1326del	Indel.	-	-	DC	Likely pathogenic	VCV001210682
		727	*p*.Leu727Pro	c.2180T > C	Missense	PDa	DA	DC	NA	NA

† indicates a novel variant in bold and a novel patient is marked with a # sign. NA indicates the items without data available. Variants marked with a hyphen are not necessary to be predicted or improper to be predicted their pathogenicity in protein level *via* SIFT, Polyphen-2; PROVEAN and MutationTaster. nonsense variants, frameshift Indel, and splicing variants may change more residues and protein length so that the position where they change cannot be predicted and is indicated by the slash in the abovementioned table. In-frame Indel may lead to deletion or insertion of amino acids and change protein length and so the position where they change is indicated by the number followed underline. Grayish lattices are the variants that have information in ClinVar and change at the same residues as where the variants identified in this study change. InDel, insertion or deletion; NA, not available; probably damaging, PDa; Da, damaging; deleterious, De; disease-causing, DC.

### *OCA2* Variants in OCA Patients

Four *OCA2* variants were detected by TES in two OCA patients (P11 and P12) and confirmed by Sanger sequencing (Figure 2). The frequency of the variants was rare or absent, and their homozygosity has not been observed in the healthy control database (Supplementary Table S1). All three missense variants (c.1001C > T, c.1325A > G, and c.2180T > C) were predicted deleterious by *in silico* analysis (Table 1). c.1001C > T has been assigned pathogenic or likely pathogenic in ClinVar but no information about other variants is realized (Table 1). The splicing variant c.808–3C > G has been confirmed pathogenic *via in vitro* splicing assay (Zhong et al., 2019). Furthermore, checking the literature reported about Chinese OCA, we found that c.1325A > G is novel and the other three variants have been reported in at least five different OCA cases in Chinese (Supplementary Table S2 and Figure 3). c.2180T > C may lead to substitution for Leu^727^ located in the permease domain (342–775aa) and in the region where the methadone tolerance landscape of *p* protein demonstrated that nearly no tolerant region locates (Wiel et al., 2019) (Supplementary Figure S3A). Therefore, c.808–3C > G can be considered likely pathogenic (PS3, PM2, and *p*P5), c.2180T > C considered likely pathogenic (PM1, PM2, and *p*P3, *p*P5), and c.1325A > G classified uncertain significance (PM2 and *p*P3).

### *In Silico* Analysis of the Impact of Novel Variants

Two novel variants NM_000372.5: c.623T > G, *p*.(Leu208Arg) and NM_000275.3: c.1325A > G, *p*.(Asn442Ser) have been found in this study. TYR sequon 202_212HEAPAFLPWHR in which Leu^208^ locates is in high homology to its paralog TYRP1 (Figure 4B), and His^202^ and His^211^ form a copper center within the catalytically active domain (Noh et al., 2020). The counterpart of Leu^208^ is Leu^60^ in tyr of *Streptomyces castaneoglobisporus* and Leu^221^ in human TYRP1. As shown by the crystal structures, Leu^60^ is inserted into the hydrophobic core formed by Phe^59^, Tyr^165^, Phe^177^, Leu^181^, Val^219,^ and Trp^223^ in Tyr (PDB: 1WX2), and Leu^221^ is inserted into the hydrophobic core formed by Phe^220^, Phe^342^, Phe^355^, Val^359^, Thr^407^, and Phe^411^ in TYRP1 (PDB: 5M8L) to stabilize the protein structure (Figures 4B,C). Leu60Arg in Tyr of *Streptomyces castaneoglobisporus* and Leu221Arg in human TYRP1 may cause steric hindrance in the proteins and introduce charges that are incompatible with the hydrophobic environment (Figures 4B,C). Alignment analysis showed that the counterparts of Phe^59^, Tyr^165^, Phe^177^, Leu^181^, Val^219,^ and Trp^223^ in tyr of *Streptomyces castaneoglobisporus* were correspondingly Phe^207^, Tyr^327^, Phe^340^, Leu^344^, Val^393^, and Phe^397^ in human TYR (Supplementary Figure S2A). The counterparts of Phe^220^, Phe^342^, Phe^355^, Val^359^, Thr^407,^ and Phe^411^ in TYRP1 were correspondingly Phe^207^, Phe^328^, Phe^340^, Leu^344^, Val^393,^ and Phe^397^ in human TYR (Supplementary Figure S2B). Therefore, we speculated that in human TYR, Leu^208^ may be inserted into the hydrophobic core which may be formed by Phe^207^, Tyr^327^, Phe^328^, Phe^340^, Leu^344^, Val^393,^ and Phe^397^. It is possible that the substitution of leucine at position 208, which has a hydrophobic side chain, by arginine, which is hydrophilic and has a positively charged side chain, may destroy hydrophobicity and affect TYR stability. In *OCA2*, the novel variant c.1325A > G identified in this study may result in the substitution of serine for Asn^442^. Asn^442^ was conserved among the orthologs of *p* protein (Supplementary Figure S3B). Structure analysis of *p* protein in AlphaFold revealed that multiple polar interactions may be formed between the side-chain of Asn442 and nearby residues Val^443^, Asn^476,^ and Asp^486^. These interactions are likely abrogated in the *p*.N442S mutant due to removal of the asparagine side-chain (Supplementary Figure S3C). Moreover, the residue Asn^442^ in sequon 442_444Asn-Val-Thr-Thr bearing canonical consensus (Asn-X-Ser/Thr-X, X≠Pro) of N-linked glycosylation is an essential posttranslational modification of secretory and membrane protein in eukaryotic cells. Moreover, the same amino acid Asn^442^ change resulting from the variant c.1324_1326del has been determined to be likely pathogenic in ClinVar (Table 1). However, further studies are necessary to verify the impact of two novel variants.

## Discussion

In this study, in total, sixteen variants were detected in twelve OCA cases including fourteen recurrent variants and two novel variants. Among the recurrent variants identified in this study, some variants such as c.929dupC and c.230_232dupGGG in *TYR* and c.808–3C > G in *OCA2* were common in OCA cases from East Asian regions, while these variants have seldom been reported in OCA cases from the Western population, even in a large cohort of 990 index patients with albinism (Lasseaux et al., 2018). On the other hand, their heterozygosity was absent in the Western population based on healthy controls of gnomAD (genome Aggregation Database) (Supplementary Table S1). In addition, a pathogenic variant *TYR*: c.896G > A is most common in Chinese OCA cases, while it is just in three of 161 TYR-related cases identified out of 990 index patients from France (Lasseaux et al., 2018). In gnomAD, we noticed that heterozygous variant c.896G > A presented in East Asians, Latino/Admixed American, and European non-Finnish and was absent in other populations (Supplementary Table S1). Hence, the abovementioned observations hinted at the possibility that the occurrence of rare variants with potential pathogenicity in Mendelian OCA genes may vary by ethnicity. In addition, three cases (P1, P3, and P4) have homozygous variants, although their parents have no consanguineous marriage. Two unrelated individuals have the same heterozygous variant due to its higher frequency in the heterozygous state, and then the offspring segregate these variants from both parents thus we speculated that a relatively higher proportion of individuals might be the carriers with heterozygous variants *TYR*: c.230G > A, *TYR*: c.346C > T or *TYR*: c.623T > C in Chinese population.

Twelve OCA cases have been identified in this study, including ten TYR-related and two OCA2-related patients. In TYR-related patients, eight patients have candidate variants in TYR within its first two exons that are mutational hotspots, while two patients (P5 and P10) have variants out of mutational hotspot. The patient P10 manifesting blond hair has been clinically diagnosed as OCA2 at first (Figure 1), and then identification of compound variants c.929dupC and c.1037–7A > T plus c.1037–10_11delTT in TYR supported to be affected by OCA1B, which is caused by the partial loss of TYR or its activity. Additionally, P12 was the adult with flaxen hair and was finally diagnosed as OCA2 due to identification of the compound variants c.2180T > C and c.1325A > G in the OCA2 gene, while most OCA2 cases have hair color from light yellow to blond or to brown. Based on the cutaneous phenotype of the cases in this study and the cases in previous reports, homozygosity or compound heterozygosity of ten variants in TYR (c.230G > A, c.230_232dupGGG, c.346C > T, c.623T > G, c.832C > T, c.895C > A, c.895C > T, c.896G > A, c.929dupC and c.1200G > T) may be related to stark white skin and white hair and no change in later age due to complete loss of melanin. Moreover, *TYR*: c.1037–7A > T plus c.1037–10_11delTT on the same allele identified in P10 may be related to creamy white skin and yellow or blond or brown hair due to residual TYR function since another allele is c.929dupC, which may bring about complete loss of TYR function. Therefore, molecular testing is conducive to a definitive diagnosis of OCA, and the identification of variants may further our understanding of the genes.

Two novel variants (*TYR*: c.623T > G and *OCA2*: c.1325A > G) were identified in this study. *TYR*: c.623T > G may lead to substitution of arginine for Leu^208^ whose counterpart is Leu^60^ in Streptomyces, and Leu^221^ in human TYRP1 may be crucial to protein stabilization as demonstrated in crystal structures (Figure 4). In addition, His^38^, His^54,^ and His^63^ in Streptomyces tyr (His^180^, His^202^, His^211^ in human TYR) coordinated a copper ion, and the sequon between His^38^ and His^63^, including Leu^60^, may be involved in the formation of the binuclear copper center as part of catalytic center (Matoba et al., 2006). The residue Leu^208^ has been mentioned for its proximity to the active site of tyrosinase and its potential role in maintaining catalytic activity (Schweikardt et al., 2007). Arginine having a positively charged side chain substituted at 208th residue for Leucine having an uncharged nonpolar side may disrupt the binuclear copper center. Also, milky skin and white hair were observed in P4 patients being a homozygosity of the variant. Hence, we speculate that Leu^208^ may also be the key residue of TYR activity. Another novel variant *OCA2*:c.1325A > G may lead to substitution of serine for Asn^442^ in *p* protein. *p* protein Structure from AlphaFold can be used for analysis, although there is no resolved crystal structure available for analysis. Although the interactions between Asn442 and nearby residues Val^443^, Asn^476,^ and Asp^486^ in *p* protein may be abrogated in the *p*.N442S mutant (Supplementary Figure S3C), the overall structure of the mutant might be unchanged since both Asn and Ser are polar amino acids. In addition, OCA2 has been suggested in a recent study to play a key role in melanosome-specific anion channel, and the putative luminal loop 440–449aa is critical to OCA2-medicated Cl^−^ current regulating melanosomal pH (Rinchik et al., 1993; Bellono et al., 2014). Sequence analysis showed that c.1325A > G in *OCA2* may disrupt a putative N-linked glycosylation site Asn^442^ that has also been predicted as a glycosylation site on the website https://www.uniprot.org/uniprot/Q04671. N-linked glycosylation is important in quality control, stability, and function of glycoproteins such as trafficking or gating kinetics of channel protein, and the sequon Asn-X-Thr/Ser-X is a key determinant in the efficiency of glycosylation (Esmail and Manolson, 2021). The variant at Val^443^ adjacent to Asn^442^ has recurred in European OCA (Hutton and Spritz, 2008; Lasseaux et al., 2018; Marti et al., 2018) and is frequently reported in Chinese OCA (Figure 3). The substitution Val443Ile near Asn^442^ has been experimentally demonstrated to reduce current amplitudes required in melanogenesis but has intact subcellular localization (Bellono et al., 2014), from which we inferred that the substitution Asn442Ser might result in dysregulation of the anion channel and impair optimal pH for TYR activity in the melanosome. The impact of these two novel variants here may deserve further study.

In summary, we reported sixteen different variants in twelve OCA cases, including two novel OCA-related variants and three cases reported for the first time based on their associated variants, which can be in favor of OCA diagnosis by expanding the phenotypic and mutational spectrum. Meanwhile, we present hair phenotypes related to two novel variants. In addition, the analysis of available crystal structures implies that Leu^208^, affected by the novel variant *TYR*: c.623T > G, may play an important role in the stability of TYR.

## Data Availability

The original contributions presented in the study are included in the article/Supplementary Material; further inquiries can be directed to the corresponding authors.
