# Supplementary material for: Identification of 12 OCA Cases in Chinese Population and Two Novel Variants
Source: Front Genet. 2022 Jul 12;13:926511. doi: 10.3389/fgene.2022.926511 (PMC9340472; doi:10.3389/fgene.2022.926511)
Supplement: Supplementary file 1 [file DataSheet1.docx]

**Identification of Twelve OCA cases in Chinese Population and Two Novel Variants**

Zilin Zhong^1-2^, Zheng Zhou^3, 4^, Jianjun Chen^1, 5, *^, Jun Zhang^6, 7, *^

**Supplementary Materials:**

1. **Table S1** Frequency of the variants in healthy controls of gnomAD.

2. **Table S2** The recurrence of variants in Chinese OCA cases.

3. **Figure S1** Confirmation of two variants of P2 on different alleles.

4. **Figure S2** Alignment of human TYR with its paralog and orthologous.

5. **Figure S3** Analysis of the residue where two VUS change in P protein.

6. **References for Table S2**

**Table S1** Frequency of the variants in healthy controls of gnomAD

| **Gene** | **rsIDs** | **AT** | **AP** |  |  |  | **Frequency** |  |  |  |  |
| --- | --- | --- | --- | --- | --- | --- | --- | --- | --- | --- | --- |
|  |  |  |  | **Total** | **EAS** | **SAS** | **AFR** | **AMR** | **ASJ** | **FIN** | **NFE** |
| *TYR* | rs1235132905 | c.929dupC | p.R311Kfs*7 | 11/251178 | 11/18386 | 0/30616 | 0/16252 | 0/34516 | 0/10070 | 0/21644 | 0/113562 |
|  | rs61754375 | c.896G>A | p.R299H | 18/282584 | 6/19936 | 0/30616 | 0/24952 | 5/35350 | 0/10360 | 0/25118 | 7/129030 |
|  | rs62645904 | c.832C>T | p.R278* | 48/282454 | 3/19944 | 39/30606 | 3/24950 | 1/35324 | 0/10360 | 0/25098 | 2/128970 |
|  | rs61753256 | c.346C>T | p.F116* | 8/282746 | 5/19952 | 1/30614 | 0/24966 | 1/35434 | 0/10362 | 0/25120 | 1/129072 |
|  | rs61753184 | c.230_232 dupGGG | p.R77_E78insG | 7/282488 | 7/19928 | 0/30602 | 0/24952 | 0/35392 | 0/10356 | 0/25108 | 0/128934 |
|  | rs61753185 | c.230G>A | p.R77Q | 25/282422 | 2/19900 | 3/30594 | 2/24950 | 1/35392 | 0/10362 | 2/25112 | 15/128894 |
|  | rs61754374 | c.895C>T | p.R299C | 4/251210 | 0/18380 | 0/30614 | 2/16252 | 0/34500 | 010072 | 0/21638 | 0/113622 |
|  | rs61754374 | c.895C>A | p.R299S | 7/251210 | 7/18380 | 0/30614 | 0/16252 | 1/34500 | 0/10072 | 0/21638 | 1/113622 |
|  | rs527352749 | c.1037-11_-10delTT | - | 5/199940 | 5/15056 | 0/23652 | 0/13796 | 0/26218 | 0/7702 | 0/15148 | 0/93636 |
|  | rs61754381 | c.1037-7T>A | - | 242/280938 | 5/19866 | 2/30530 | 2/24946 | 15/35208 | 155/10330 | 1/24300 | 45/128602 |
|  | rs145610977 | c.1200G>T | p.W400C | 3/250294 | 0/18384 | 2/30606 | 0/16166 | 0/34408 | 0/10066 | 0/21596 | 1/112980 |
|  | / | c.623T>G^†^ | p.L208R | / | / | / | / | / | / | / | / |
| *OCA2* | rs1169783208 | c.808-3C>G | - | 1/251448 | 1/18394 | 0/30614 | 0/16254 | 0/34592 | 0/10080 | 0/21614 | 0/113760 |
|  | rs121918168 | c.1001C>T | p.A334V | 2/251086 | 0/18386 | 1/30604 | 0/16228 | 0/34570 | 0/10074 | 0/21502 | 1/113594 |
|  | / | c.1325A>G^†^ | p.N442S | / | / | / | / | / | / | / | / |
|  | / | c.2180T>C | p.L727P | / | / | / | / | / | / | / | / |

**Note**: Chr, Chromosome; AT, Alternatives in Transcript sequence; AP, Alternatives in Protein sequence; AC, Allele Count; AN, Allele Number; HC, Homozygote Count. EAS, East Asian; SAS, South Asian; AFR, African/ African-American; AMR, Latino/ Admixed American; ASJ, Ashkenazi Jewish; FIN, European (Finnish); NFE, European non-Finnish. The sign ^†^ indicates a novel variant; the protein change is hard to be predicated marked with hyphen in AP; slash indicates the items without data in gnomAD.

| **Table S2** The recurrence of variants in Chinese OCA cases. | | | | | | | |
| --- | --- | --- | --- | --- | --- | --- | --- |
| **Gene** | **AA where variants change** | **Allele 1** | **Allele 2** | **Status** | **Cutaneous phenotype** | **References** |  |
| TYR | 311- | c.929dupC,  p.(Arg311Lysfs*7) | c.929dupC,p.(Arg311Lysfs*7) | HOM. | white skin, white hair | Wang et al, 2009;Wei et al, 2010;Wei et al, 2011;Wei et al, 2015[2];Sun et al, 2018;Zhong et al, 2019[4];Lin et al, 2019;Yang et al, 2019;Chuan et al, 2020[7] |  |
|  |  |  | c.896G>A,p.(Arg299His) | HET. | white skin, white hair and no change with age | Wang et al, 2009;Wei et al, 2010[4];Wei et al, 2011[5];Liu et al, 2014[2];Wei et al, 2015[5];Sun et al, 2018;Zhong et al, 2019[4];Luo et al, 2019;Lin et al, 2019[3];Yang et al, 2019;Chuan et al, 2020[2];Power,2019;**This study** |  |
|  |  |  | c.832C>T,p.(Arg278*) | HET. | white skin, white hair or blond hair | Li et al, 2006;Wang et al, 2009;Wei et al, 2010[5];Wu et al, 2012;Wei et al, 2015[2];Wang et al, 2016[2];Qiu et al, 2018;Zhong et al, 2019;Lin et al, 2019;Luo et al, 2019;Chuan et al, 2020[3] |  |
|  |  |  | c.346C>T,p.(Arg116*) | HET. | white skin, white hair and no change with age | Wei et al, 2010;Liu et al, 2014;Zhong et al, 2019;Luo et al, 2019 |  |
|  |  |  | c.230_232dupGGG,p.(Arg77_Glu78insGly) | HET. | white skin, white hair and no change with age | Wei et al, 2010;Lin et al, 2014;Wei et al, 2015[2];Qiu et al, 2018;Zhong et al, 2019 |  |
|  |  |  | c.230G>A,p.(Arg77Gln) | HET. | white skin, white hair | Wei et al, 2010[2];Zhong et al, 2019[2];Luo et al, 2019[2];Chuan et al, 2020 |  |
|  |  |  | c.895C>A,p.(Arg299Ser) | HET. | white skin, white hair | Xu et al, 2021 |  |
|  |  |  | c.1037-7T>A,c.1037-10_11delTT | HET. | white skin, yellow or blond or pale brown hair | Lin et al, 2014;Liu et al, 2014;**This study** |  |
|  |  |  | c.895C>T,p.(Arg299Cys) | HET. | OCA1A | Wei et al, 2010;**This study** |  |
|  |  |  | c.229C>G,p.(Arg77Gly) | HET. | OCA1 | Zhong et al, 2019 |  |
|  |  |  | c.1037-2A>T | HET. | OCA1 | Zhong et al, 2019 |  |
|  |  |  | c.1199G>T,p.(Trp400Leu) | HET. | white skin, white hair | Wei et al, 2010[3];Zheng et al, 2011[2];Wu et al, 2012; Zhong et al, 2019;Luo et al, 2019;Chuan et al, 2020 |  |
|  |  |  | c.1A>G,p.(Met1?) | HET. | OCA1A | Zhong et al, 2019 |  |
|  |  |  | c.47C>T,p.(Ser16Phe) | HET. | white skin, white hair and no change with age | Wang et al, 2020 |  |
|  |  |  | c.71G>A,p.(Cys24Tyr) | HET. | OCA | Wei et al, 2015[2] |  |
|  |  |  | c.157G>T,p.(Gly53Cys) | HET. | white skin, white hair | Chuan et al, 2020[2]; |  |
|  |  |  | c.164G>A,p.(Cys55Tyr) | HET. | white skin, white hair | Chuan et al, 2020[3] |  |
|  |  |  | c.216del A,p.(Val74Trpfs*46) | HET. | white skin, white hair | Chuan et al, 2020 |  |
|  |  |  | c.298T>C,p.(Cys100Arg) | HET. | white skin, yellow white hair | Chuan et al, 2020 |  |
|  |  |  | c.324G>A,p.(Trp108*) | HET. | OCA | Zhong et al, 2019 |  |
|  |  |  | c.334T>C,p.(Cys112Arg) | HET. | white skin, white hair and no change in later age | Wei et al, 2015 |  |
|  |  |  | c.425A>T,p.(Lys142Met) | HET. | OCA | Zhong et al, 2019 |  |
|  |  |  | c.561_562insTTATTATGTGTCAAATTATCCCCCA,p.(Gly190Cysfs*12) | HET. | white skin, white hair | Liu et al,2014;Zhong et al, 2019 |  |
|  |  |  | c.575C>A,p.(Ser192Tyr) | HET. | OCA | Wei et al, 2015 |  |
|  |  |  | c.640_642delTTC ,p.(Phe214del) | HET. | OCA | Liu et al, 2010 |  |
|  |  |  | c.655G>A,p.(Glu219Lys) | HET. | white skin, white hair | Wei et al, 2015;Zhong et al, 2019;Luo et al, 2019 |  |
|  |  |  | c.714G>A,p.(Trp238*) | HET. | OCA1A | Wei et al, 2011 |  |
|  |  |  | c.757G>T,p.(Gly253*) | HET. | white skin, white hair and no change in later age | Wang et al, 2020 |  |
|  |  |  | c.758G>A,p.(Gly253Glu) | HET. | OCA1B | Wei et al, 2010 |  |
|  |  |  | c.867C>A,p.(Cys289*) | HET. | white skin, white hair | Xu et al, 2021 |  |
|  |  |  | c.902C>G,p.(Pro301Arg) | HET. | white skin, white hair and darken with age | Wang et al, 2020 |  |
|  |  |  | c.575C>A,p.(Ser192Tyr) | HET. | OCA1B | Wei et al, 2010 |  |
|  |  |  | c.944C>G,p.(Ser315*) | HET. | OCA | Zhong et al, 2019 |  |
|  |  |  | c.1021A>G,p.(Arg341Gly) | HET. | white skin, white hair | Lin et al, 2019 |  |
|  |  |  | c.1037G>A,p.(Gly346Glu) | HET. | OCA | Zhong et al, 2019 |  |
|  |  |  | c.1088A>G,p.(His363Arg) | HET. | white skin, white hair | Luo et al, 2019 |  |
|  |  |  | c.1146C>G,p.(Asn382Lys) | HET. | OCA1A | Wei et al, 2010 |  |
|  |  |  | c.1168C>G,p.(His390Asp) | HET. | OCA1A | Wei et al, 2010 |  |
|  |  |  | c.1195C>T,p.(Gln399*) | HET. | white skin, yellow white hair | Chuan et al, 2020 |  |
|  |  |  | c.1204C>T,p.(Arg402*) | HET. | pink white skin, white hair | Zhong et al, 2019;Xu et al, 2021 |  |
|  |  |  | c.1205G>A,p.(Arg402Gln) | HET. | white skin, white hair | Chuan et al, 2020 |  |
|  |  |  | c.1258C>T,p.(His420Tyr) | HET. | white skin, white hair and no change with age | Wang et al, 2020 |  |
|  |  |  | c.1265G>A,p.(Arg422Gln) | HET. | white skin, white hair | Wei et al, 2010;Zhong et al, 2019;Chuan et al, 2020 |  |
|  |  |  | c.1366G>C,p.(Asp456His) | HET. | white skin, white hair and no change with age | Wang et al, 2020 |  |
|  |  |  | c.1425G>A,p.(Trp475*) | HET. | white skin, white hair | Zhong et al, 2019;Chuan et al, 2020 |  |
|  |  |  | c.1447A>G,p.(Met483Val) | HET. | white skin, white hair and no change with age | Wang et al, 2020 |  |
|  | 299 | c.896G>A,  p.(Arg299His) | c.896G>A,p.(Arg299His) | HOM. | white skin, white or light yellow or blond hair | Liu et al, 2010;Wei et al, 2010[4];Wei et al, 2015[2];Lu et al, 2017;Qiu et al, 2018;Zhong et al, 2019[5];Yang et al, 2019;Luo et al, 2019[3];Xu et al, 2021[2]; |  |
|  |  |  | c.832C>T,p.(Arg278*) | HET. | white skin, white or yellow hair and no change with age/OCA1A | Wei et al, 2010[3];Wei et al, 2011[3];Lin et al, 2014;Liu et al, 2014[2];Wei et al, 2015[2];Luo et al, 2019;Zhong et al, 2019;Chuan et al, 2020[3];Xu et al, 2021;**This study** |  |
|  |  |  | c.346C>T,p.(Arg116*) | HET. | white skin, white or yellow hair and no change with age | Wei et al, 2011;Zheng et al, 2011;Wu et al, 2012;Liu et al, 2014[2];Lin et al, 2014;Zhong et al, 2019[2];Chuan et al, 2020 |  |
|  |  |  | c.230_232dupGGG,  p.(Arg77_Glu78insGly) | HET. | white skin, white hair and no change with age | Wei et al, 2010;Zheng et al, 2011;Wu et al, 2012;Liu et al, 2014;Wei et al, 2015;Qiu et al, 2018;Zhong et al, 2019[2];Power et al, 2019 |  |
|  |  |  | c.230G>A,p.(Arg77Gln) | HET. | white skin, white hair and no change with age | Wei et al, 2010;Wei et al, 2011;Lin et al, 2014;Liu et al, 2014 |  |
|  |  |  | c.895C>A,p.(Arg299Ser) | HET. | white skin, white hair | Wei et al, 2011;Lin et al, 2014;Xu et al, 2021 |  |
|  |  |  | c.1037-7T>A,c.1037-10_11delTT | HET. | OCA2 like | Wei et al, 2010[2];Zhong et al, 2019 |  |
|  |  |  | c.895C>T,p.(Arg299Cys) | HET. | OCA1 | Wei et al, 2015;Zhong et al, 2019 |  |
|  |  |  | c.229C>T,p.(Arg77Trp) | HET. | white skin, white hair | Luo et al, 2019;Chuan et al, 2020 |  |
|  |  |  | c.229C>G,p.(Arg77Gly) | HET. | OCA1A | Zhong et al, 2019 |  |
|  |  |  | c.1198T>G,p.(Trp400Gly) | HET. | OCA | Sun et al, 2018 |  |
|  |  |  | c.1198T>A,p.(Trp400Arg) | HET. | white skin, white hair | Luo et al, 2019 |  |
|  |  |  | c.1199G>T,p.(Trp400Leu) | HET. | white skin, white hair/OCA1A | Wei et al, 2010;Zheng et al, 2011[2];Lin et al, 2014;Wei et al, 2015;Zhong et al, 2019[2];Chuan et al, 2020 |  |
|  |  |  | c.1A>G,p.(Met1?) | HET. | OCA | Sun et al, 2018 |  |
|  |  |  | c.56A>G,p.(His19Arg) | HET. | OCA1A | Wei et al, 2010 |  |
|  |  |  | c.70T>C,p.(Cys24Arg) | HET. | OCA1A | Wei et al, 2010;Sun et al, 2018 |  |
|  |  |  | c.71G>A,p.(Cys24Tyr) | HET. | white skin, white hair | Chuan et al, 2020[2] |  |
|  |  |  | c.117G>T,p.(Trp39Cys) | HET. | OCA1A | Wei et al, 2010 |  |
|  |  |  | c.157G>T,p.(Gly53Cys) | HET. | white skin, white hair and no change with age | Wang et al, 2020 |  |
|  |  |  | c.164G>A,p.(Cys55Tyr) | HET. | white skin, white hair | Luo et al, 2019 |  |
|  |  |  | c.300T>G,p.(Cys100Trp) | HET. | OCA1A | Wei et al, 2010 |  |
|  |  |  | c.425A>T,p.(Lys142Met) | HET. | white skin, light yellow hair/OCA1B | Wang et al, 2009;Wei et al, 2010 |  |
|  |  |  | c.549_550delGT,p.(Ser184Asnfs*9) | HET. | white skin, white hair | Wang et al, 2015 |  |
|  |  |  | c.633T>A,p.(His211Gln) | HET. | white skin, white hair and no change with age | Wang et al, 2020 |  |
|  |  |  | c.650G>C,p.(Arg217Pro) | HET. | white skin, white hair and no change with age | Wang et al, 2020 |  |
|  |  |  | c.653G>A,p.(Trp218*) | HET. | OCA1 | Zhong et al, 2019 |  |
|  |  |  | c.655G>A,p.(Glu219Lys) | HET. | white skin, white hair | Wei et al, 2015,power et al,2019 |  |
|  |  |  | c.703T>C,p.(Tyr235His) | HET. | white skin, white hair | Zhong et al, 2019;Chuan et al, 2020 |  |
|  |  |  | c.758G>A,p.(Gly253Glu) | HET. | white skin, white hair and no change with age | Wei et al, 2011;Liu et al, 2014;Sun et al, 2018 |  |
|  |  |  | c.820-3C>G | HET. | OCA1 | Zhong et al, 2019[2]; |  |
|  |  |  | c.1106A>G,p.(Tyr369Cys) | HET. | white skin, white hair | Lin et al, 2019 |  |
|  |  |  | c.1121T>C,p.(Met374Thr) | HET. | white skin, white hair and no change with age | Wang et al, 2020 |  |
|  |  |  | c.1196delA,p.(Gln399Argfs*86) | HET. | OCA1A | Wei et al, 2011 |  |
|  |  |  | c.1195C>T,p.(Gln399*) | HET. | white skin, white hair and no change with age | Wu et al, 2012;Liu et al, 2014 |  |
|  |  |  | c.1205G>A,p.(Arg402Gln) | HET. | white skin, white hair | Chuan et al, 2020 |  |
|  |  |  | c.1217C>A,p.(Pro406Leu) | HET. | OCA1A | Wei et al, 2010 |  |
|  |  |  | c.1265G>A,p.(Arg422Gln) | HET. | white skin, light yellow hair | Wei et al, 2011;Luo et al, 2019 |  |
|  |  |  | c.1337G>T,p.(Gly446Val) | HET. | white skin, white hair and no change with age | Liu et al, 2014 |  |
|  |  |  | c.1425G>A,p.(Trp475*) | HET. | white skin, white hair | Qiu et al, 2018 |  |
|  |  |  | c.1445_1446insTCCTTGGGGCGGC,p.(Met483Profs*31) | HET. | white skin, white hair and no change with age | Wang et al, 2020 |  |
|  | 278- | c.832C>T,  p.(Arg278*) | c.832C>T,p.(Arg278*) | HOM. | white skin, white hair/OCA1B | Wei et al, 2010;Wei et al, 2011;Wei et al, 2015;Lin et al, 2019;Chuan et al, 2020 |  |
|  |  |  | c.346C>T,p.(Arg116*) | HET. | white skin, white hair and no change with age | Wei et al, 2010;Lin et al, 2014;Liu et al, 2014;Sun et al, 2018;Lin et al, 2019 |  |
|  |  |  | c.230_232dupGGG,p.(Arg77_Glu78insGly) | HET. | OCA1A | Wei et al, 2010 |  |
|  |  |  | c.230G>A,p.(Arg77Gln) | HET. | white skin, white hair and no change with age | Wu et al, 2012;Liu et al, 2014 |  |
|  |  |  | c.895C>A,p.(Arg299Ser) | HET. | white skin, white hair and no change with age/OCA1A | Wang et al, 2009;Wei et al, 2010;**This study** |  |
|  |  |  | c.1037-7T>A,c.1037-10_11delTT | HET. | OCA2 like | Wei et al, 2010;Wei et al, 2011 |  |
|  |  |  | c.1199G>T,p.(Trp400Leu) | HET. | white skin, white hair | Wei et al, 2010;Wei et al, 2015;Qiu et al, 2018;Zhong et al, 2019;Luo et al, 2019[2] |  |
|  |  |  | c.71G>A,p.(Cys24Tyr) | HET. | white skin, white hair | Wei et al, 2011;Lin et al, 2014;Wei et al, 2015 |  |
|  |  |  | c.107G>C,p.(Cys36Ser) | HET. | pink white skin, white hair | Chuan et al, 2020 |  |
|  |  |  | c.157G>T,p.(Gly53Cys) | HET. | white skin, white hair | Luo et al, 2019 |  |
|  |  |  | c.164G>A,p.(Cys55Tyr) | HET. | white skin, white hair | Wei et al, 2010;Lin et al, 2014 |  |
|  |  |  | c.425A>T,p.(Lys142Met) | HET. | white skin, white hair and no change with age | Liu et al, 2014 |  |
|  |  |  | c.454C>T,p.(Pro152His) | HET. | white skin, white hair and no change with age | Wei et al, 2015 |  |
|  |  |  | c.655G>A,p.(Glu219Lys) | HET. | OCA1 | Zhong et al, 2019[2] |  |
|  |  |  | c.706T>C,p.(Trp236Arg) | HET. | OCA1A | Wei et al, 2010 |  |
|  |  |  | c.758G>A,p.(Gly253Glu) | HET. | white skin, white hair | Chuan et al, 2020 |  |
|  |  |  | c.820-3C>G | HET. | light yellow hair/OCA1B | Wang et al, 2009;Wei et al, 2010 |  |
|  |  |  | c.880G>A,p.(Glu294Lys) | HET. | OCA1A | Zheng et al, 2011 |  |
|  |  |  | c.985T>C,p.(Ser329Pro) | HET. | white skin, white hair | Wang et al, 2015 |  |
|  |  |  | c.1088A>G,p.(His363Arg) | HET. | white skin, white hair | Qiu et al, 2018 |  |
|  |  |  | c.1168C>G,p.(His390Asp) | HET. | OCA1 | Wei et al, 2015;Zhong et al, 2019 |  |
|  |  |  | c.1195C>T,p.(Gln399*) | HET. | white skin, white hair | Luo et al, 2019 |  |
|  |  |  | c.1211A>C,p.(His404Pro) | HET. | OCA1A | Wei et al, 2010[2] |  |
|  |  |  | c.1265G>A,p.(Arg422Gln) | HET. | white skin, white hair | Qiu et al, 2018 |  |
|  |  |  | c.1275C>G,p.(Tyr425*) | HET. | white skin, white hair and no change with age | Wang et al, 2020 |  |
|  | 116 | c.346C>T,  p.(Arg116*) | c.346C>T,p.(Arg116*) | HOM. | white skin, white hair and no change with age/OCA1A | Wang et al, 2009;Wei et al, 2010[2];Zheng et al, 2011;Yang et al, 2019;**This study** |  |
|  |  |  | c.230_232dupGGG,p.(Arg77_Glu78insGly) | HET. | OCA1A | Zheng et al, 2011 |  |
|  |  |  | c.895C>A,p.(Arg299Ser) | HET. | OCA1 | Zhong et al, 2019 |  |
|  |  |  | c.1200G>T,p.(Trp400Cys) | HET. | white skin, white hair and no change with age | **This study^#^** |  |
|  |  |  | c.229C>T,p.(Arg77Trp) | HET. | OCA1 | Zhong et al, 2019 |  |
|  |  |  | c.1037-7T>A | HET. | white skin, white hair | Chuan et al, 2020 |  |
|  |  |  | c.1037-2A>T | HET. | white skin, white hair | Xu et al, 2021 |  |
|  |  |  | c.1199G>T,p.(Trp400Leu) | HET. | white skin, white hair and no change with age | Zheng et al, 2011[2];Liu et al, 2014;Lin et al, 2019 |  |
|  |  |  | c.71G>A,p.(Cys24Tyr) | HET. | white skin, white hair/OCA1A | Wei et al, 2010;Wang et al, 2009;Wei et al, 2015;Chuan et al, 2020 |  |
|  |  |  | c.164G>A,p.(Cys55Tyr) | HET. | white skin, white hair and no change with age | Wei et al, 2010 |  |
|  |  |  | c.456delC,p.(Ile153*) | HET. | OCA1 | Zhong et al, 2019 |  |
|  |  |  | c.819G>C,p.(Gln273His) | HET. | OCA1 | Zhong et al, 2019 |  |
|  |  |  | c.985T>C,p.(Ser329Pro) | HET. | white skin, white hair | Qiu et al, 2018 |  |
|  |  |  | c.1169A>G,p.(His390Arg) | HET. | OCA1 | Zhong et al, 2019 |  |
|  |  |  | c.1184+2T>C | HET. | white skin, white hair and no change with age | Liu et al, 2014 |  |
|  |  |  | c.1204C>T,p.(Arg402*) | HET. | OCA1 | Zhong et al, 2019 |  |
|  |  |  | c.1366+1delG | HET. | white skin, white hair and no change with age | Liu et al, 2014 |  |
|  |  |  | c.1425G>A,p.(Trp475*) | HET. | white skin, white hair | Wei et al, 2010 |  |
|  | 77- | c.230_232dupGGG,  p.(Arg77_Glu78insGly) | c.230_232dupGGG,p.(Arg77_Glu78insGly) | HOM. | OCA1 | Wei et al, 2015;Zhong et al, 2019[2] |  |
|  |  |  | c.230G>A,p.(Arg77Gln) | HET. | white skin, white and no change with age | Sun et al, 2018;Luo et al, 2019;Chuan et al, 2020;**This study** |  |
|  |  |  | c.895C>A,p.(Arg299Ser) | HET. | white skin, light yellow hair | Xu et al, 2021[2]; |  |
|  |  |  | c.1037-7T>A,c.1037-10_11delTT | HET. | white skin, white hair at birth and brown or yellow hair in later age | Liu et al, 2014[3]; |  |
|  |  |  | c.1200G>A,p.(Trp400*) | HET. | white skin, white hair | Chuan et al, 2020 |  |
|  |  |  | c.1037-10_11delTT | HET. | white skin, white hair | Wu et al, 2012 |  |
|  |  |  | c.1037-7T>A | HET. | white skin, light yellow hair | Sun et al, 2018;Luo et al, 2019 |  |
|  |  |  | c.1199G>T,p.(Trp400Leu) | HET. | white skin, white hair and no change with age | Wei et al, 2010;Liu et al, 2014;Zhong et al, 2019 |  |
|  |  |  | c.1A>G,p.(Met1?) | HET. | white skin, white hair and no change with age | Liu et al, 2014 |  |
|  |  |  | c.71G>A,p.(Cys24Tyr) | HET. | OCA1A | Wei et al, 2010 |  |
|  |  |  | c.840G>T,p.(Glu281*) | HET. | white skin, white hair and no change with age | Wang et al, 2020 |  |
|  |  |  | c.1088A>G,p.(His363Arg) | HET. | white skin, white hair and no change with age | Wei et al, 2015 |  |
|  |  |  | c.1147G>A,p.(Asp383Asn) | HET. | OCA1B | Wei et al, 2010 |  |
|  |  |  | c.1204C>T,p.(Arg402*) | HET. | OCA1A | Wei et al, 2010 |  |
|  |  |  | c.1233T>A,p.(Tyr411*) | HET. | white skin, white hair and no change with age | Wang et al, 2020 |  |
|  |  |  | c.1234C>A,p.(Pro412Thr) | HET. | OCA | Zhong et al, 2019 |  |
|  |  |  | c.1235C>T,p.(Pro412Leu) | HET. | white skin, white hair | Power et al, 2019 |  |
|  | 77 | c.230G>A,  p.(Arg77Gln) | c.230G>A,p.(Arg77Gln) | HOM. | white skin, white hair and no change with age /OCA1A | Yang et al, 2019;**This study** |  |
|  |  |  | c.229C>T,p.(Arg77Trp) | HET. | OCA1B | Wei et al, 2010 |  |
|  |  |  | c.1199G>T,p.(Trp400Leu) | HET. | white skin, white hair and no change with age | Zheng et al, 2011;Liu et al, 2014;Zhong et al, 2019;Yang et al, 2019 |  |
|  |  |  | c.71G>A,p.(Cys24Tyr) | HET. | white skin, white hair/OCA1A | Wang et al, 2009;Wei et al, 2010[2] |  |
|  |  |  | c.126C>A,p.(Asp42GLu) | HET. | white skin, white hair and no change with age | Wang et al, 2020 |  |
|  |  |  | c.1092T>G,p.(Asn364Lys) | HET. | white skin, white hair | Lin et al, 2014 |  |
|  |  |  | c.1146C>G,p.(Asn382Lys) | HET. | OCA | Wei et al, 2015 |  |
|  |  |  | c.1204C>T,p.(Arg402*) | HET. | OCA1 | Zhong et al, 2019 |  |
|  |  |  | c.1277T>A,p.(Met426 Lys) | HET. | OCA2 like | Wei et al, 2011;Wei et al, 2015 |  |
|  |  | c.229C>G,  p.(Arg77Gly) | c.229C>G,p.(Arg77Gly) | HOM. | OCA1 | Zhong et al, 2019 |  |
|  |  |  | c.1146C>G,p.(Asn382Lys) | HET. | OCA2 like | Wei et al, 2010 |  |
|  |  | c.229C>T,  p.(Arg77Trp) | c.715C>T,p.(Arg239Trp) | HET. | OCA1 | Zhong et al, 2019 |  |
|  | 299 | c.895C>A,  p.(Arg299Ser) | c.24C>A,p.(Cys8*) | HET. | OCA1 | Zhong et al, 2019 |  |
|  |  |  | c.71G>A,p.(Cys24Tyr) | HET. | OCA | Wei et al, 2015 |  |
|  |  |  | c.229C>T,p.(Arg77Trp) | HET. | white skin, white hair | Xu et al, 2021 |  |
|  |  |  | c.731G>A,p.(Cys244Tyr) | HET. | white skin, white hair | Xu et al, 2021[2]; |  |
|  |  |  | c.1516C>T,p.(Gly506*) | HET. | OCA1 | Wei et al, 2010 |  |
|  | / | c.1037-7T>A,  c.1037-10_11delTT | c.655G>A,p.(Glu219Lys) | HET. | white skin, white hair at birth and yellow hair in later age | Liu et al, 2014 |  |
|  |  |  | c.815G>A,p.(Trp272*) | HET. | OCA | Wei et al, 2015 |  |
|  |  |  | c.1255G>A,p.(Gly419Arg) | HET. | OCA2 like | Wei et al, 2010;Wei et al, 2015 |  |
|  |  |  | c.1114delG,p.(Gly372fs*112) | HET. | milky skin, white hair | Wang et al, 2015 |  |
|  | 299 | c.895C>T,  p.(Arg299Cys) | c.895C>T,p.(Arg299Cys) | HOM. | white skin, white hair and no change with age | Liu et al, 2014 |  |
|  |  |  | c.1199G>T,p.(Trp400Leu) | HET. | white skin, light yellow hair | Liu et al, 2010[2];Zhong et al, 2019;Lin et al, 2019 |  |
|  | 400 | c.1200G>T,  p.(Trp400Cys) | c.1199G>T,p.(Trp400Leu) | HET. | white skin, white hair and no change with age | Wang et al, 2020 |  |
|  |  | c.1199G>T,  p.(Trp400Leu) | c.1199G>T,p.(Trp400Leu) | HOM. | white skin, white hair | Luo et al, 2019[2];Xu et al, 2021 |  |
|  |  |  | c.56A>G,p.(His19Arg) | HET. | white skin, white hair | Lin et al, 2014;Wei et al, 2015 |  |
|  |  |  | c.71G>A,p.(Cys24Tyr) | HET. | OCA2 like | Wei et al, 2010 |  |
|  |  |  | c.164G>A,p.(Cys55Tyr) | HET. | white skin, white hair | Qiu et al, 2018 |  |
|  |  |  | c.425A>T,p.(Lys142Met) | HET. | OCA1A | Zheng et al, 2011 |  |
|  |  |  | c.636A>T,p.(Arg212Ser) | HET. | OCA1 | Zhong et al, 2019 |  |
|  |  |  | c.709G>T,p.(Asp237Tyr) | HET. | white skin, white hair and no change with age | Wang et al, 2020 |  |
|  |  |  | c.758G>A,p.(Gly253Glu) | HET. | white skin, white hair and no change with age | Liu et al, 2014;Qiu et al, 2018;Zhong et al, 2019[2] |  |
|  |  |  | c.820-3C>G | HET. | OCA1A | Zheng et al, 2011 |  |
|  |  |  | c.1136G>T,p.(Gly379Val) | HET. | OCA | Wei et al, 2015 |  |
|  |  |  | c.1168C>G,p.(His390Asp) | HET. | white skin, white hair and no change with age | Lin et al, 2014 |  |
|  | 208 | c.623T>G,  p.(Leu208Arg)^†^ | c.623T>G,p.(Leu208Arg)^†^ | HOM. | white skin, white hair and no change with age | **This study**^#^ |  |
| *OCA2* | / | c.808-3C>G | c.808-3C>G | HOM. | white skin, light yellow hair/OCA2 | Tan et al, 2019;Zhong et al, 2019;Yang et al, 2019 |  |
|  |  |  | c.1001C>T,p.(Ala334Val) | HET. | white skin, yellow or golden hair, darken with age | Chuan et al, 2020; **This study** |  |
|  |  |  | c.171delG,p.(Gln58fs*44) | HET. | white skin, white hair and no change with age | Wang et al, 2020 |  |
|  |  |  | c.632C>T,p.(Pro211Leu) | HET. | white skin, light yellow hair/OCA2 | Tan et al, 2019;Yang et al, 2019,[2] |  |
|  |  |  | c.1363A>G,p.(Arg455Gly) | HET. | OCA2 | Yang et al, 2019 |  |
|  |  |  | c.1441G>A,p.(Ala481Thr) | HET. | OCA | Ma et al, 2021 |  |
|  |  |  | c.1832T>C,p.(Leu611Pro) | HET. | White skin, light yellow hair/OCA2 | Yang et al, 2019, [3]; Tan et al, 2019 |  |
|  |  |  | c.2080-2A>G | HET. | milky skin, yellow hair | Wang et al, 2017 |  |
|  |  |  | c.2272G>C,p.(Asp758His) | HET. | white skin, white hair and no change with age | Wang et al, 2020 |  |
|  |  |  | c.2363C>T,p.(Ser788Leu) | HET. | OCA2 | Yang et al, 2019 |  |
|  | 334 | c.1001C>T,  p.(Ala334Val) | c.515+1delG | HET. | white skin, white hair and no change with age | Wang et al, 2020 |  |
|  |  |  | c.1349C>T,p.(Thr450Met) | HET. | OCA2 | Wei et al, 2011;Wei et al, 2015 |  |
|  |  |  | c.1832T>C,p.(Leu611Pro) | HET. | OCA2 | Yang et al, 2019 |  |
|  |  |  | c.2139G>A,p.(Lys713Lys)[affect splicing] | HET. | white skin, white hair | Qiu et al, 2018 |  |
|  | 727 | c.2180T>C,  p.(Leu727Pro) | c.1325A>G,p.(Asn442Ser)^†^ | HET. | creamy white skin, flaxen hair, darken with age, freckles in face | **This study**^#^ |  |
|  |  |  | c.1327G>A, p.(Val443Ile) | HET. | OCA2 | Yang et al, 2019 |  |
|  |  |  | c.406C>T,p.(Arg136*) | HET. | white skin, golden yellow hair | Luo et al,2019 |  |
|  |  |  | c.808-2A>G | HET. | OCA | Hu et al, 2014 |  |
|  |  |  | c.1255C>T,p.(Arg419Trp) | HET. | white skin, white hair | Luo et al,2019;Ma et al, 2021 |  |
|  |  |  | c.1349C>T,p.(Thr450Met) | HET. | OCA2 | Wei et al, 2010 |  |
|  |  |  | c.1714C>T,p.(Arg572Cys) | HET. | OCA1B like | Yang et al, 2019 |  |
|  | 443 | c.1327G>A,  p.(Val443Ile) | c.1139_1141delTTG, p.(Val380del) | HET. | OCA2 | Yang et al, 2019 |  |
|  |  |  | c.1363A>G, p.(Arg455Gly) | HET. | OCA1A like | Yang et al, 2019 |  |
|  |  |  | c.1423A>C, p.(Thr475Pro) | HET. | white skin, light yellow hair/OCA2 | Tan et al, 2019, Yang et al, 2019 |  |
|  |  |  | c.1832T>C, p.(Leu611Pro) | HET. | OCA2 | Yang et al, 2019 |  |
|  |  |  | c.1970G>T, p.(Gly657Val) | HET. | white skin, white hair | Qiu et al,2018 |  |
|  |  |  | c.2228C>T, p.(Pro743Leu) | HET. | white skin, light yellow hair | Tan et al, 2019 |  |
|  |  |  | c.2323G>C,p.(Gly775Arg) | HET. | white skin, light yellow hair | Luo et al, 2019 |  |
|  |  |  | c.2360C>T,p.(Ala787Val) | HET. | white skin, light yellow or flaxen hair | Zhang 2013;Wei et al,2015;Tan et al, 2019 |  |
| **Note**: The table presents the reported OCA cases in Chinese population based on the literatures (till 31 October, 2021) in PubMed and its links. Based on phenotype, the cases with stark white skin and white hair were generally diagnosed them as OCA1; If the history of no change in cutaneous color with age were provided, these cases with white skin and white hair were diagnosed as OCA1A; the cases with white skin, white hair at birth and darken in later age were diagnosed them as OCA1B; the cases with creamy white skin (not stark white like OCA1) and yellow/blond/light brown/brown hair were generally diagnosed as OCA2. And the cases with conspicuous hypopigmentation in skin and hair have been recorded as OCA. The lattices in dark gray are the variants that are different from the variants identified in this study. Light grayish lattices are the variants which have information in ClinVar and change at the same residues as the variants identified in this study. AA, amino acid; HET., heterozygote; HOM., homozygote; EX., Exon; INV, intron. Novel cases are indicated by the sign # after the words "This study" and novel variants by the sign †. | | | | | | | |


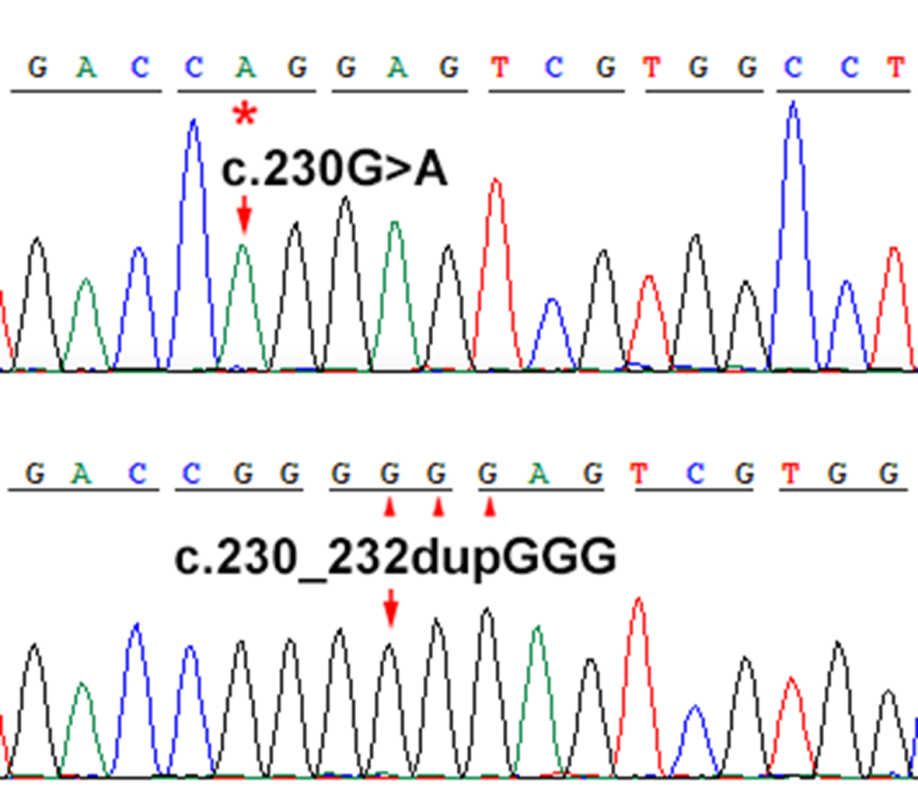


**Figure S1 Confirmation of two *TYR* variants on different alleles of P2.** The upper is the sequence of the clone with the amplicon of the variant c.230G>A on one allele and the lower is the sequence of the clone with the amplicon of the variant c.230_232dupGGG on another allele. These two variants were confirmed to locate on different alleles of P2. c.230G>A was pointed out by a red star and three red triangles indicated the insertion of every G. The variants were pointed out in each of sequence chromatograms.


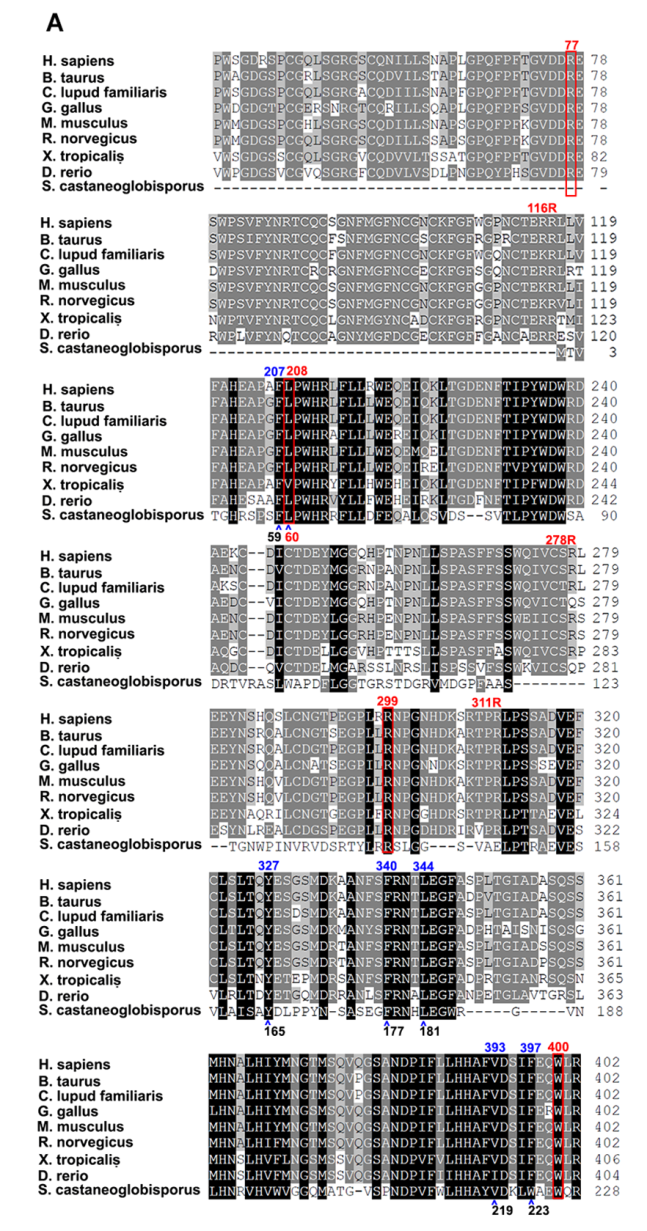


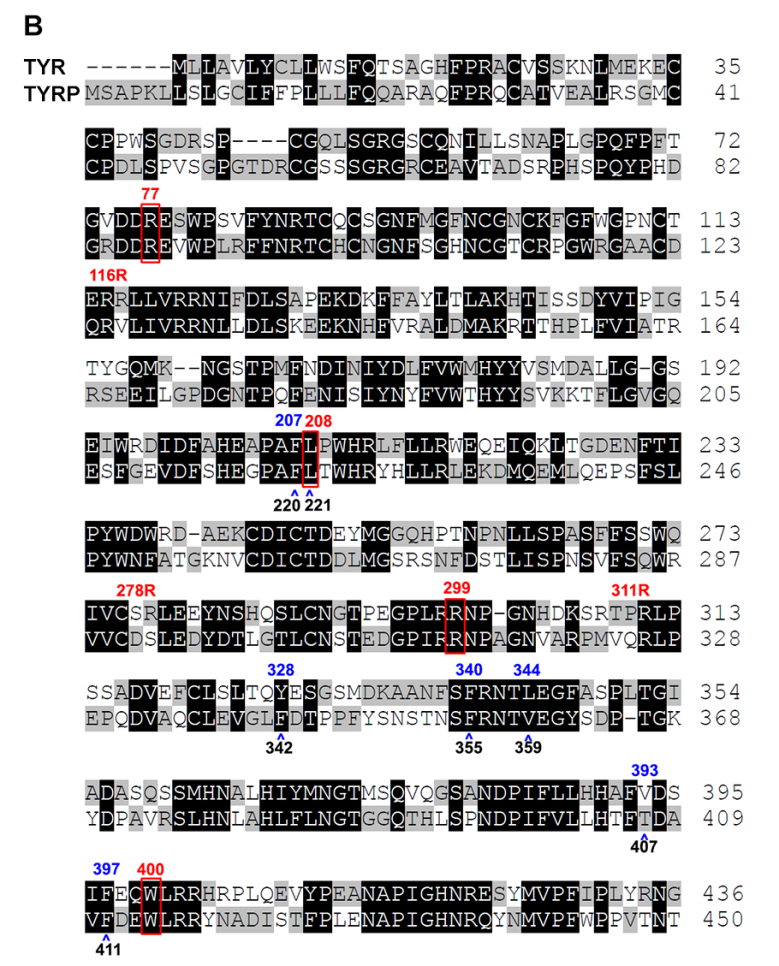


**Figure S2** Alignment of human TYR with its paralog and orthologous. Part of sequence where variants changes and relevant sequence are shown to evaluate the potential impact that variants may bring about. The numbers in red indicate the positions where *TYR* variants in this study change. The numbers in red indicate the positions where variants in this study changes. Amino acids in red box show there is high conservation at residues where the missense substitutes or in-frame InDel (Insertions or Deletions) changes. The number with amino acids in red shows the positions of residues where the nonsense changes or frameshift InDel starts to change. Part of sequence where variants changes and relevant sequence are shown to evaluate the potential impact that variants may bring about. The numbers in red indicate the positions where *TYR* variants in this study change. Amino acids in red box shown there is high conservation at residues where the missense substitute or in-frame InDel changes. The number with amino acids in red shows the positions of residues where the nonsense changes or frameshift InDel starts to change. (A) Evolutionary conservation of TYR was analyzed via alignment between Homo Sapiens TYR and its orthologous. The numbers in black with blue head of open arrow are the position of the residues in tyr of Streptomyces castaneoglobisporus shown in Figure 4B and their counterparts in human TYR are indicated in the numbers in blue. (B) Alignment between TYR and TYRP1 of Homo sapiens shows that both of them are highly homologous. The numbers in black with blue head of open arrow head are the position of the residues in TYRP1 shown in Figure 4C and their counterparts in human TYR are indicated in the numbers in blue.


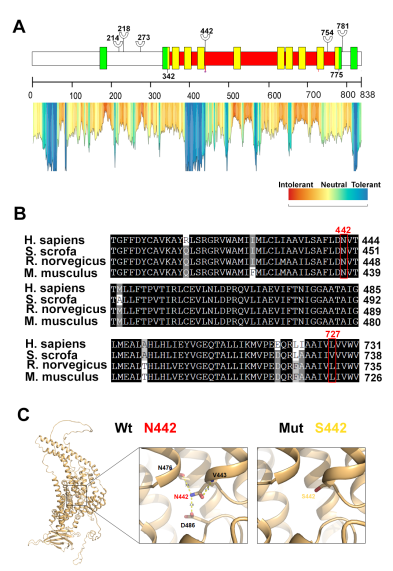


**Figure S3 Analysis of the residues where two VUS change in P protein.** (A) The schematic representation and tolerance landscape of P protein. In the schematics, P_permease domain (342-775aa) is in red; transmembrane domains out of P_permease domain are in green, transmembrane domains within P_permease domain are in yellow. Branch symbols indicate putative N-linked glycosylation sites. The residues where two VUS change are pointed by red lines including the novel variant pointed by a red arrow head plus a purple blob. (B) Alignment of human P protein with its orthologous. Highly conserved residues among the species that has P protein of access number heading with NP here are shaded with black. The positions of amino acid that VUS change are indicated in red digitals. N^442^ or L^727^ in human P protein and its counterpart are in the red frame. (C) Residue N^442^ in human P protein and the effect of substitution N442S. Left square: Structural analysis of wildtype P protein. It reveals multiple polar interactions between N^442^ side-chain and its nearby residues V^443^, N^476^, D^486^ in P protein. Right square: Structural analysis of its N442S mutant. These interactions are likely abrogated in N442S mutant due to the removal of side-chain of asparagine.

**References for Table S2**

Chuan, Z., Yan, Y., Hao, S., Zhang, Q., Zhou, B., Feng, X., et al. (2021). Mutation Analysis of 63 Northwest Chinese Probands with Oculocutaneous Albinism. *Curr Eye Res* 46(1)**,** 140-143. doi: 10.1080/02713683.2020.1781192.

Hu, H., Wang, H., Jia, Z., and Xie, Q. (2014). [Prenatal genetic diagnosis for two Chinese families affected with oculocutaneous albinism type ]. *Zhonghua Yi Xue Yi Chuan Xue Za Zhi* 31(4)**,** 424-427. doi: 10.3760/cma.j.issn.1003-9406.2014.04.003.

Li, H.Y., Wu, W.I., Zheng, H., Duan, H.L., Chen, Z., and Chen, L.M. (2006). [Prenatal gene diagnosis of oculocutaneous albinism type I]. *Zhonghua Yi Xue Yi Chuan Xue Za Zhi* 23(3)**,** 280-282.

Lin, Y., Chen, X., Yang, Y., Che, F., Zhang, S., Yuan, L., et al. (2019). Mutational Analysis of TYR, OCA2, and SLC45A2 Genes in Chinese Families with Oculocutaneous Albinism. *Mol Genet Genomic Med* 7(7)**,** e00687. doi: 10.1002/mgg3.687.

Lin, Y.Y., Wei, A.H., He, X., Zhou, Z.Y., Lian, S., and Zhu, W. (2014). A comprehensive study of oculocutaneous albinism type 1 reveals three previously unidentified alleles on the TYR gene. *Eur J Dermatol* 24(2)**,** 168-173. doi: 10.1684/ejd.2014.2304.

Liu, J., Choy, K.W., Chan, L.W., Leung, T.Y., Tam, P.O., Chiang, S.W., et al. (2010). Tyrosinase gene (TYR) mutations in Chinese patients with oculocutaneous albinism type 1. *Clin Exp Ophthalmol* 38(1)**,** 37-42. doi: 10.1111/j.1442-9071.2009.02220.x.

Liu, N., Kong, X.D., Shi, H.R., Wu, Q.H., and Jiang, M. (2014). Tyrosinase gene mutations in the Chinese Han population with OCA1. *Genet Res (Camb)* 96**,** e14. doi: 10.1017/S0016672314000160.

Lu, Q., Yuan, L., Xu, H., Huang, X., Yang, Z., Yi, J., et al. (2017). Identification of a missense mutation in the tyrosinase gene in a Chinese family with oculocutaneous albinism type 1. *Mol Med Rep* 15(3)**,** 1426-1430. doi: 10.3892/mmr.2017.6137.

Luo, D., Linpeng, S., Zeng, L., Tan, H., Li, Z., and Wu, L. (2019). Molecular genetic study of 59 Chinese Oculocutaneous albinism families. *Eur J Med Genet* 62(10)**,** 103709. doi: 10.1016/j.ejmg.2019.103709.

Ma, L., Zhu, J., Wang, J., Huang, Y., Zhang, J., Wang, C., et al. (2021). Genetic Analysis of 28 Chinese Families With Tyrosinase-Positive Oculocutaneous Albinism. *Front Genet* 12**,** 715437. doi: 10.3389/fgene.2021.715437.

Power, B., Ferreira, C.R., Chen, D., Zein, W.M., O'Brien, K.J., Introne, W.J., et al. (2019). Hermansky-Pudlak syndrome and oculocutaneous albinism in Chinese children with pigmentation defects and easy bruising. *Orphanet J Rare Dis* 14(1)**,** 52. doi: 10.1186/s13023-019-1023-7.

Qiu, B., Ma, T., Peng, C., Zheng, X., and Yang, J. (2018). Identification of Five Novel Variants in Chinese Oculocutaneous Albinism by Targeted Next-Generation Sequencing. *Genet Test Mol Biomarkers* 22(4)**,** 252-258. doi: 10.1089/gtmb.2017.0211.

Sun, W., Shen, Y., Shan, S., Han, L., Li, Y., Zhou, Z., et al. (2018). Identification of TYR mutations in patients with oculocutaneous albinism. *Mol Med Rep* 17(6)**,** 8409-8413. doi: 10.3892/mmr.2018.8881.

Tan, J., Pan, L., Huang, J., Li, W., Li, Z., Chang, R., et al. (2019). [Analysis of P gene variations among fourteen patients with oculocutaneous albinism type II]. *Zhonghua Yi Xue Yi Chuan Xue Za Zhi* 36(12)**,** 1163-1166. doi: 10.3760/cma.j.issn.1003-9406.2019.12.003.

Wang, C., Chen, C., Zhao, X., Zhao, G., Liu, L., and Kong, X. (2020). [Spectrum of pathological genetic variants among 405 Chinese pedigrees affected with oculocutaneous albinism]. *Zhonghua Yi Xue Yi Chuan Xue Za Zhi* 37(7)**,** 725-730. doi: 10.3760/cma.j.issn.1003-9406.2020.07.006.

Wang, X., Zhu, Y., Shen, N., Peng, J., Wang, C., Liu, H., et al. (2016). Mutation analysis of a Chinese family with oculocutaneous albinism. *Oncotarget* 7(51)**,** 84981-84988. doi: 10.18632/oncotarget.13109.

Wang, X., Zhu, Y., Shen, N., Peng, J., Wang, C., Liu, H., et al. (2017). Mutational analysis of a Chinese family with oculocutaneous albinism type 2. *Oncotarget* 8(41)**,** 70345-70355. doi: 10.18632/oncotarget.19697.

Wang, Y., Guo, X., Li, W., and Lian, S. (2009). Four novel mutations of TYR gene in Chinese OCA1 patients. *J Dermatol Sci* 53(1)**,** 80-81. doi: 10.1016/j.jdermsci.2008.07.002.

Wang, Y., Wang, Z., Chen, M., Fan, N., Yang, J., Liu, L., et al. (2015). Mutational Analysis of the TYR and OCA2 Genes in Four Chinese Families with Oculocutaneous Albinism. *PLoS One* 10(4)**,** e0125651. doi: 10.1371/journal.pone.0125651.

Wei, A., Wang, Y., Long, Y., Wang, Y., Guo, X., Zhou, Z., et al. (2010). A comprehensive analysis reveals mutational spectra and common alleles in Chinese patients with oculocutaneous albinism. *J Invest Dermatol* 130(3)**,** 716-724. doi: 10.1038/jid.2009.339.

Wei, A., Yang, X., Lian, S., and Li, W. (2011). Implementation of an optimized strategy for genetic testing of the Chinese patients with oculocutaneous albinism. *J Dermatol Sci* 62(2)**,** 124-127. doi: 10.1016/j.jdermsci.2011.02.009.

Wei, A.H., Zang, D.J., Zhang, Z., Yang, X.M., and Li, W. (2015). Prenatal genotyping of four common oculocutaneous albinism genes in 51 Chinese families. *J Genet Genomics* 42(6)**,** 279-286. doi: 10.1016/j.jgg.2015.05.001.

Wu, Q., Shi, H.R., Liu, N., Lu, N., Jiang, M., Zhao, Z.H., et al. (2012). [Early prenatal genetic diagnosis of oculocutaneous albinism type I in seven families]. *Zhonghua Yi Xue Yi Chuan Xue Za Zhi* 29(4)**,** 377-381. doi: 10.3760/cma.j.issn.1003-9406.2012.04.001.

Xu, C., Xiang, Y., Li, H., Xu, Y., Mao, Y., Zhou, L., et al. (2021). Genetic analysis and prenatal diagnosis of 20 Chinese families with oculocutaneous albinism. *J Clin Lab Anal* 35(2)**,** e23647. doi: 10.1002/jcla.23647.

Yang, Q., Yi, S., Li, M., Xie, B., Luo, J., Wang, J., et al. (2019). Genetic analyses of oculocutaneous albinism types 1 and 2 with four novel mutations. *BMC Med Genet* 20(1)**,** 106. doi: 10.1186/s12881-019-0842-7.

Zhang, L., Xu, B., Zhong, Y., Chen, X., Zheng, H., Jiang, W., et al. (2013). [A de novo mutation of P gene causes oculocutaneous albinism type 2 with prenatal diagnosis]. *Zhonghua Yi Xue Yi Chuan Xue Za Zhi* 30(3)**,** 318-321. doi: 10.3760/cma.j.issn.1003-9406.2013.03.015.

Zheng, H., Huang, Z.G., Wen, R.Q., and Li, H.Y. (2011). [Study of tyrosinase gene mutation in oculocutaneous albinism type 1 patients]. *Zhongguo Ying Yong Sheng Li Xue Za Zhi* 27(3)**,** 329-332.

Zhong, Z., Gu, L., Zheng, X., Ma, N., Wu, Z., Duan, J., et al. (2019). Comprehensive analysis of spectral distribution of a large cohort of Chinese patients with non-syndromic oculocutaneous albinism facilitates genetic diagnosis. *Pigment Cell Melanoma Res* 32(5)**,** 672-686. doi: 10.1111/pcmr.12790.
